# Data on preparation and characterization of an insect odorant receptor based biosensor

**DOI:** 10.1016/j.dib.2018.11.018

**Published:** 2018-11-07

**Authors:** Roshan Khadka, Nihan Aydemir, Colm Carraher, Cyril Hamiaux, Damon Colbert, Jamal Cheema, Jenny Malmström, Andrew Kralicek, Jadranka Travas-Sejdic

**Affiliations:** a Polymer Electronic Research Centre, School of Chemical Sciences, University of Auckland, Auckland 1023, New Zealand; b MacDiarmid Institute for Advanced Materials and Nanotechnology, Wellington 6140, New Zealand; c The New Zealand Institute for Plant and Food Research Limited, Private Bag 92169, Auckland 1142, New Zealand; d Department of Chemical and Materials Engineering, University of Auckland, Auckland 1023, New Zealand

## Abstract

Insect Odorant receptors (OrXs) can be used as the recognition element in a biosensor as they demonstrate high levels of sensitivity and selectivity towards volatile organic compounds. Herein, we describe a method to express and purify insect odorant receptors and reconstitute them into artificial lipid bilayers (liposomes). These OrX/liposomes were covalently attached to a gold surface and characterized using quartz crystal microbalance with dissipation monitoring (QCM-D). The interaction of OrX/liposomes immobilized on a gold surface to positive and negative odorants were studied by means of electrochemical impedance spectroscopy (EIS) and QCM-D. The data presented in this article are related to the research article titled “An ultrasensitive electrochemical impedance-based biosensor using insect odorant receptors to detect odorants” [1].

**Specifications table**

**Table:** 

Subject area	*Chemistry, Material Science*
More specific subject area	*Biosensors*
Type of data	*Table, graph, and figure*
How data was acquired	QCM-D graphs (*Q-sense analyzer (Biolin Scientific)), EIS graphs (PalmSens potentiostat)*
Data format	*Analyzed*
Experimental factors	*Gold electrodes functionalization, and electrical properties of OrX/liposome immobilization and ligand binding.*
Experimental features	*OrX/liposomes were covalently attached to gold surface and QCM-D and EIS studies were utilized to investigate ligand-binding.*
Data source location	*The University of Auckland, Auckland, New Zealand*
Data accessibility	*Data are presented in this article*
Related research article	*Khadka, R., Aydemir, N., Carraher, C., Hamiaux, C., Colbert, D., Cheema, J., Malmström, J., Kralicek, A., Travas-Sejdic, J.,* 2018 [1].

**Value of the data**

• The method used here to purify OrX subunits and integrating into lipid bilayers of liposomes to provide a platform for biosensing studies.
• The QCM-D data after covalent attachment of OrX/liposomes on the gold surface followed by the binding of ligand is very useful to understand the mechanism of signal transduction.
• The EIS capacitance data can be used to compare the sensitivity of this novel insect OrX based biosensor with other relevant OrX based biosensors.

## 1 Data

**Fig. 1:**
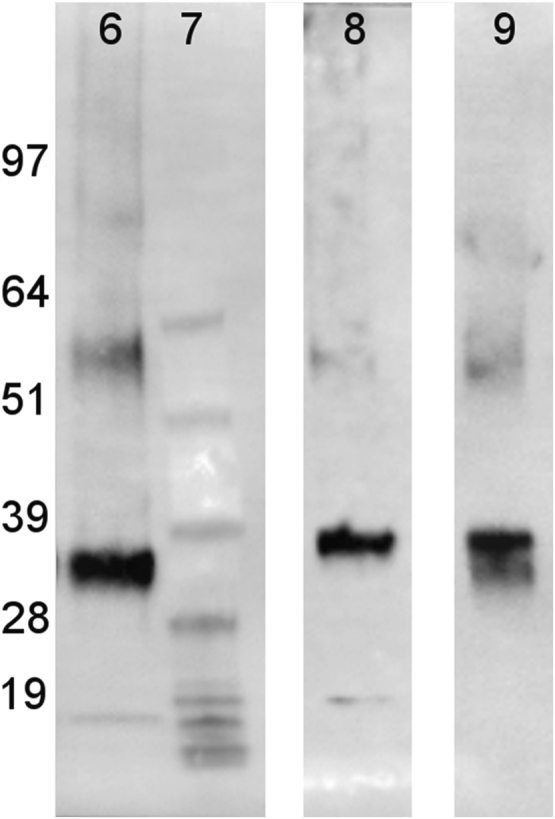
Western Blot analysis of OrX/liposomes. Lane 6: Or71a/liposomes (Anti-FLAG). Lane 7: Molecular weight marker. Lane 8: Or10a/liposomes (Anti-FLAG). Lane 9: Or22a/liposomes (Anti-FLAG). Molecular weight of the markers (kDa) are indicated.

**Fig. 2:**
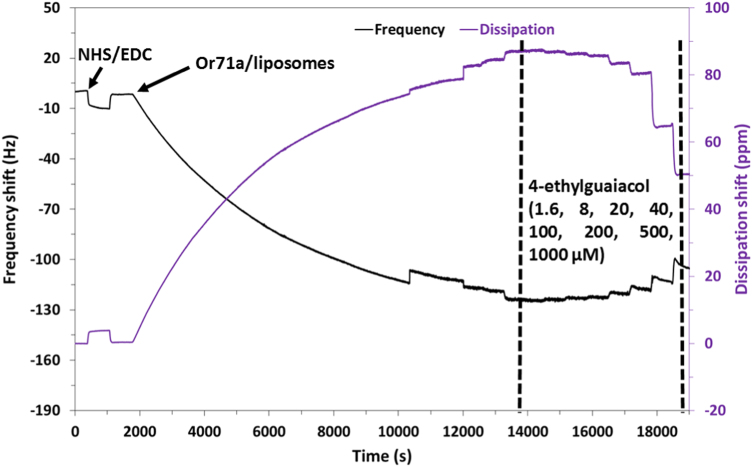
Change in frequency and dissipation of a gold surface with self-assembled monolayers (SAMs) of 6-mercaptohexanoic acid (MHA) and N-hydroxysuccinimide (NHS)/1-ethyl-3-(3-dimethylaminopropyl)-carbodiimide) (EDC) modification, followed by Or71a/liposome immobilization on the QCM sensor and then binding of the target ligand 4-ethylguaiacol.

**Fig. 3:**
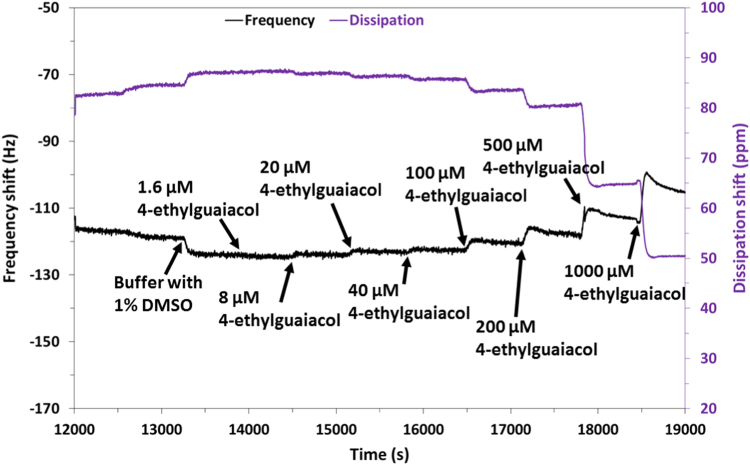
Close up view of the change in frequency and dissipation with increasing concentrations of 4-ethylguaiacol (0, 1.6, 8, 20, 40, 100, 200, 500 and 1000 µM) for the Or71a/liposome QCM sensor.

**Fig. 4:**
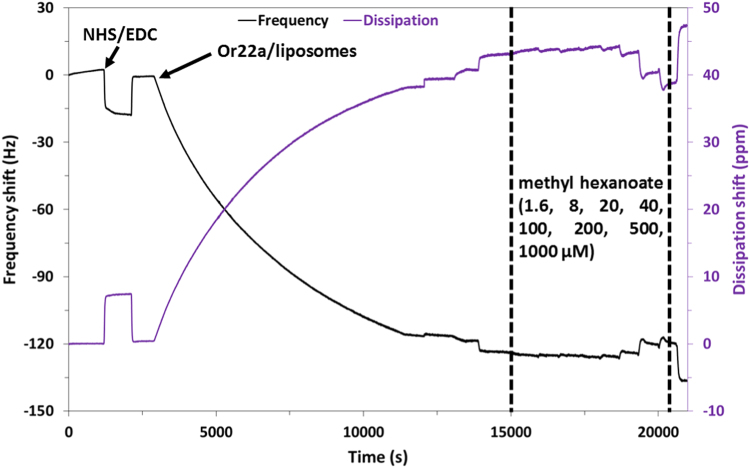
Change in frequency and dissipation of gold surface with SAM and NHS/EDC modification, followed by Or22a/liposome immobilization on the QCM sensor and then binding of the target ligand methyl hexanoate.

**Fig. 5:**
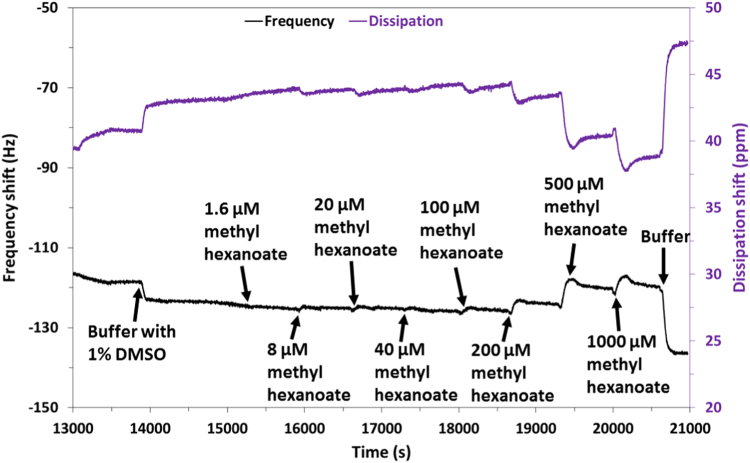
Close up view of the change in frequency and dissipation with increasing concentrations of methyl hexanoate (0, 1.6, 8, 20, 40, 100, 200, 500 and 1000 µM) for the Or22a/liposome QCM sensor.

**Fig. 6:**
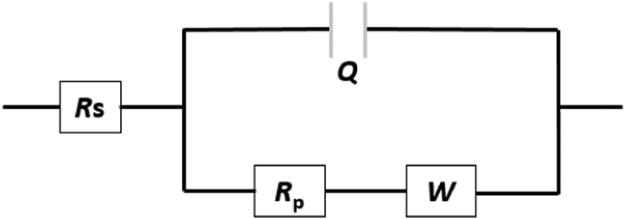
Equivalent circuit diagram (Randles model) for fitting the non-Faradaic impedance curves where *R*s is solution resistance, *Q* refers to capacitance, *R*_p_ is called polarization resistance and *W* refers to Warburg diffusion.

**Fig. 7:**
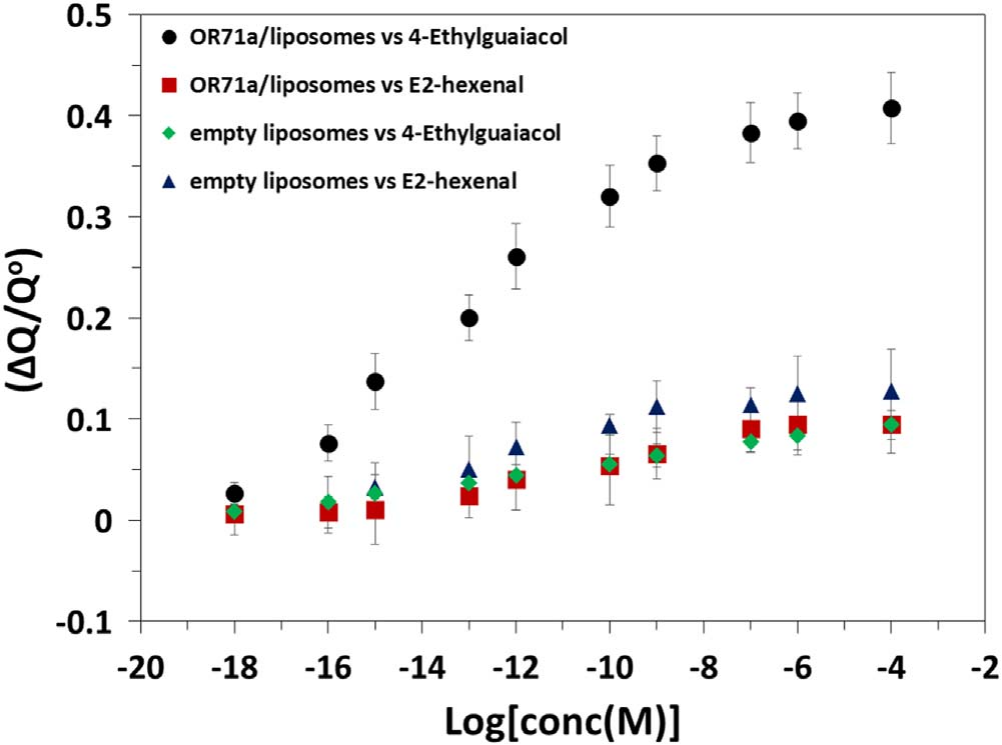
Dose response curve in terms of change in normalized capacitance values for the Or71a/liposome EIS based sensor showing detection of the specific ligand 4-ethylguaiacol with high selectivity and sensitivity. Error bars; standard deviation (SD) were generated using four repeats.

**Fig. 8:**
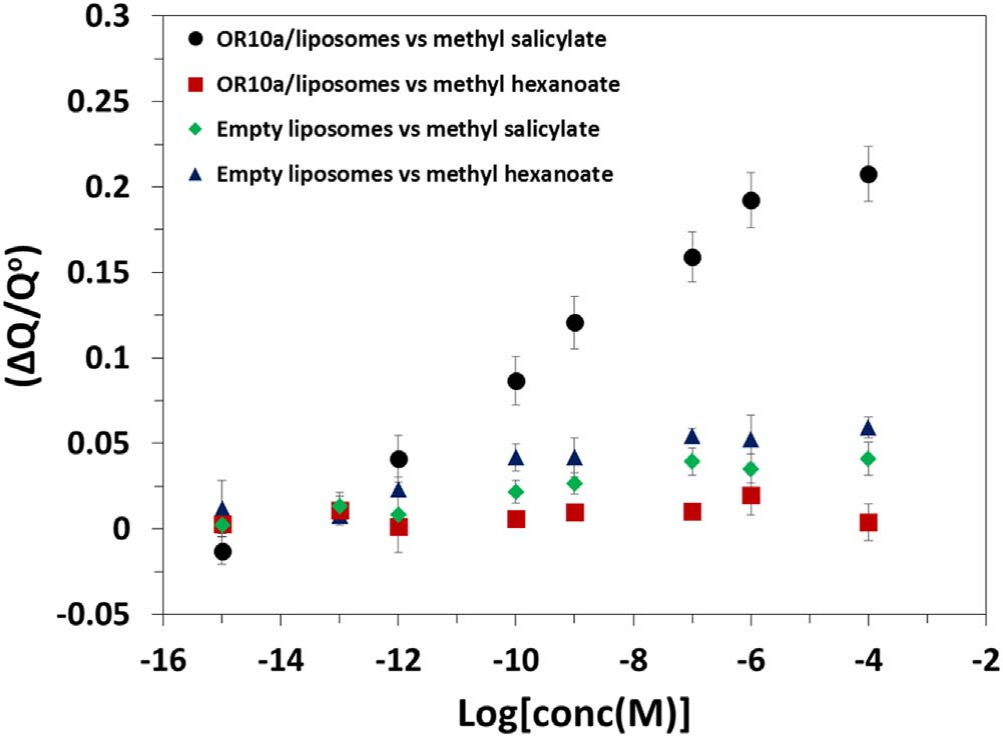
Dose response curve in terms of change in normalized capacitance values for Or10a/liposomes on an EIS based sensor showing detection of its specific ligand methyl salicylate with high selectivity and sensitivity. Error bars; standard deviation (SD) were generated using four repeats.

**Fig. 9:**
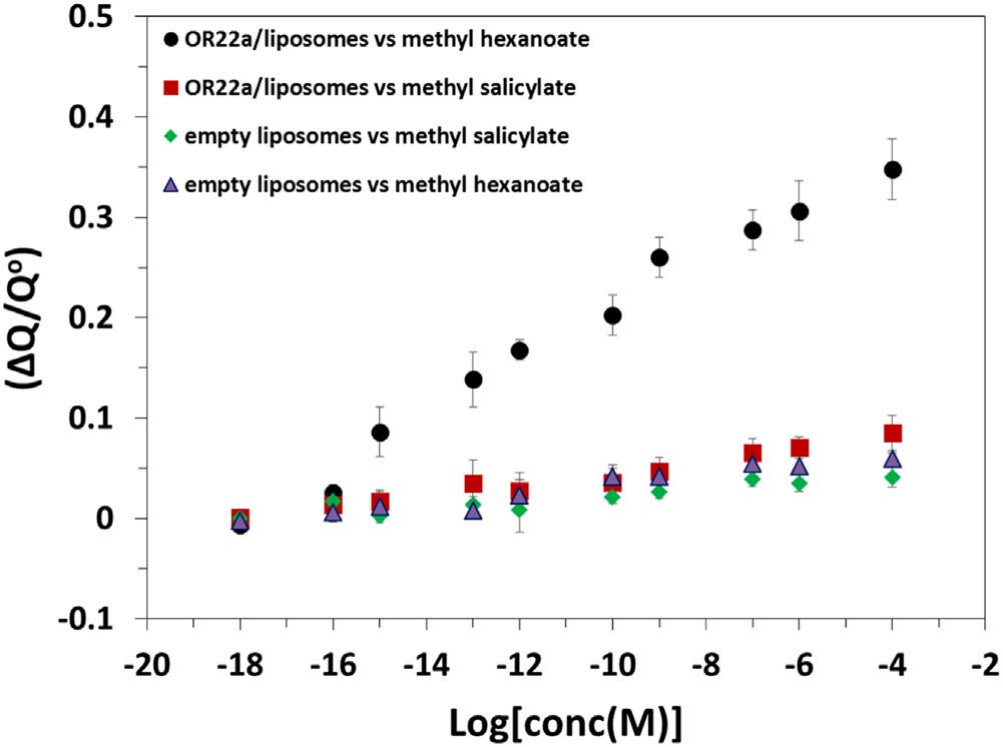
Dose response curve in terms of change in normalized capacitance values for Or22a/liposomes on an EIS based sensor showing detection of its specific ligand methyl hexanoate with high selectivity and sensitivity. Error bars; standard deviation (SD) were generated using four repeats.

The western blot analysis of purified OrX subunits are shown in Fig. 1. QCM-D data showing the real time covalent attachment of Or22a/liposomes and Or71a/liposomes on gold sensors followed by the binding of methyl hexanoate and 4-ethylguaiacol respectively, Fig. 2, Fig. 3, Fig. 4, Fig. 5. The equivalent electrical circuit model used for fitting the impedance data is shown in Fig. 6 and the calibration plots in terms of change in normalized capacitance values for three EIS sensors (Or10a, Or22a, and Or71a) are presented in Fig. 7, Fig. 8, Fig. 9.

## 2 Experimental design, materials, and methods

### 2.1 Preparation of purified OrX subunits

The purification procedure is a variation on the one detailed in Carraher et al. [2]. To his-tag affinity purify recombinantly expressed OrX subunits from baculovirus-infected Sf9 cells, 500 mL of Sf9 cells at a concentration of at 2 × 10^6^ mL^−1^ (measured with a haemocytometer), were infected with baculovirus at a multiplicity of infection (MOI) of 0.1, and incubated at 27 °C for 72 h with shaking at 110 RPM. The cell pellet was collected by centrifugation at 3,800 g for 10 min at room temperature and then resuspended in 40 mL of resuspension buffer A (20 mM Tris/HCl pH 7.5, 100 mM NaCl, 1x protease inhibitor cocktail (Roche Diagnostics GmbH, Germany)), with 25 U/mL Benzonase, then lysed by two passes on an Emulsiflex C5 emulsifier (Avestin, Germany) at 10,000–15,000 psi. The sample was then centrifuged at 1,000 g for 5 min to remove whole cells and nuclei. The supernatant was removed and spun at 100,000 g for 1 h at 4 °C. The membrane pellet was resuspended in 40 mL of buffer A with 1% w/v detergent (Zwittergent 3–16) and rotated for 1 h at room temperature at 10 rpm. The sample was then centrifuged at 100,000 g for 1 h at 18 °C. The supernatant was removed and loaded onto a 1-mL NiNTA column (GE Healthcare) where the zwittergent 3–16 detergent was exchanged to Fos-Choline 14 (FC-14). The column was washed in ten column volumes of buffer B (20 mM Tris/HCl pH 7.5, 0.36 mM FC-14) with 300 mM NaCl and 20 mM imidazole, and a further ten column volumes of buffer B with 100 mM NaCl and 50 mM imidazole. Protein was eluted with 4 column volumes of buffer B with 100 mM NaCl and 500 mM imidazole. Purity was assessed on Coomassie stained SDS–PAGE gels and by Western blotting.

Purification was completed with a final size exclusion chromatography (SEC) step. The elution fractions from the NiNTA purification were pooled and centrifuged at 20,000 g for 5 min to remove aggregates and contaminants. Then 5 mL of sample was injected onto a Superdex 200 16/60 column (GE Healthcare) attached to an Akta-Pure chromatography system (GE Healthcare). The sample was run at 1 mL/min in buffer B with 100 mM NaCl, and 2 mL fractions were collected and concentrated using a 100 kDa MWCO Vivaspin filter unit (Sartorius, Goettingen Germany) and stored at −80 °C.

### 2.2 Preparation of liposome integrated OrX subunits

Liposomes were prepared as outlined in Carraher et al. [2].

### 2.3 EIS and QCM-D measurements

For QCM-D experiments, 100 nm thick gold sensor crystals were functionalized with self-assembled monolayers (SAMs) of 6-mercapto hexanoic acid (MHA) by incubating them in MHA solution overnight. MHA functionalized crystals were placed in the QCM-D chamber and real time measurements of NHS/EDC activation followed by binding of the OrX/liposomes was monitored. Different concentrations of positive and negative odorants were flowed into the chamber and changes in frequency (∆f) and dissipation (∆D) values were recorded.

For EIS experiments, a gold disk as a working electrode, platinum coil as counter electrode and Ag/AgCl (3 M NaCl, 0.209 V vs. SHE) as a reference electrode was used. EIS measurements were performed at a fixed voltage of −0.7 using a PalmSens potentiostat with degassed PBS solution as an electrolyte.
